# Spinal infection caused by *Aspergillus flavus* in a diabetic: a case report and literature review

**DOI:** 10.3389/fmed.2024.1348203

**Published:** 2024-02-02

**Authors:** Hongtao Li, Hongyu Pan, Yang Lei, Haozhong Wang, Sen Li, Changming Xiao

**Affiliations:** ^1^Department of Spinal Surgery, The Affiliated Traditional Chinese Medicine Hospital, Southwest Medical University, Luzhou, China; ^2^Division of Spine Surgery, Department of Orthopedic Surgery, Nanjing Drum Tower Hospital, Affiliated Hospital of Medical School, Nanjing University, Nanjing, China

**Keywords:** spinal infection, *Aspergillus flavus*, fungi, voriconazole, terbinafine hydrochloride, diabetes

## Abstract

Spinal infections, notably those induced by *Aspergillus flavus* (*A. flavus*), represent a complex and uncommon clinical challenge. In individuals with diabetes mellitus, the risk is exacerbated due to a compromised immune response and a heightened vulnerability to non-standard pathogens. This case report chronicles the intricate diagnostic and treatment journey of a 59-year-old diabetic patient grappling with a spinal infection attributed to *A. flavus*. The diagnosis was delayed due to non-specific symptoms and unclear radiological signs. The administration of voriconazole, a targeted antifungal treatment, resulted in a significant clinical and radiological improvement, underscoring its effectiveness in treating such unusual fungal spinal infections; meanwhile, we found that terbinafine hydrochloride also has a similar effect in treating fungal spinal infections. This case underscores the importance of considering fungal causes in spinal infections among diabetic patients and highlights prompt diagnosis and individualized targeted antifungal therapy.

## Introduction

Spinal infections can be caused by a range of pathogens, including bacteria, such as *Staphylococcus aureus* and fungi like *Aspergillus flavus* (*A. flavus*) (1). Although bacterial infections are more prevalent, they generally allow for easier and more standardized early diagnosis and treatment. However, fungal spinal infections, particularly those caused by *A. flavus*, present unique challenges due to their complexity and severity (2). This is especially true in immunocompromised individuals, including those with diabetes mellitus. *A. flavus*, known for its invasive nature, not only significantly impacts spinal health, often leading to conditions such as spondylodiscitis, but also affects other systems including the skin and respiratory and visceral organs (3, 4). The diagnosis of such infections is often hindered by non-specific symptoms and necessitates invasive diagnostic measures, including biopsies and surgeries (5). This challenge is exacerbated in diabetic patients, where *Aspergillus* is difficult to detect due to its typically low concentration and immune compromise related to diabetes (6). Currently, there is no standard or unified clinical treatment strategy for diabetic patients with fungal spinal infections. In diabetic patients, the risk of various infections, particularly rare fungal types, is heightened due to complications like microvascular disease and neuropathy, which often mask infection symptoms and delay treatment (7). This underscores the need for swift and effective diagnosis and treatment of spinal infections in diabetic patients, highlighting their complexity and urgency. Here, we report a case of spinal infection in a diabetic patient caused by *A. flavus*.

## Case presentation

A 59-year-old male sanitation worker was admitted on March 7, 2022, presenting with a year-long history of lower back pain, leg pain, and numbness, which had worsened over the past month. The patient, a type 2 diabetic with irregular metformin adherence, had previously sought pain relief through long-term acupuncture and oral NSAIDs, and had a history of COVID-19 infection. Physical examination revealed pronounced tenderness and percussion pain from the L3 to L5 vertebral bodies, a strength of grade III in the right knee extensor, and positive bilateral Lasegue’s signs. Neurological assessment indicated diminished superficial sensation in both lower limbs. Laboratory findings showed anemia (Hemoglobin: 92 g/L), neutrophilia (NEUT%: 85%), raised inflammatory markers (ESR: 96 mm/H, CRP: 51.11 mg/L), and osteoporosis (T-score: −2.95).

**Figure 1:**
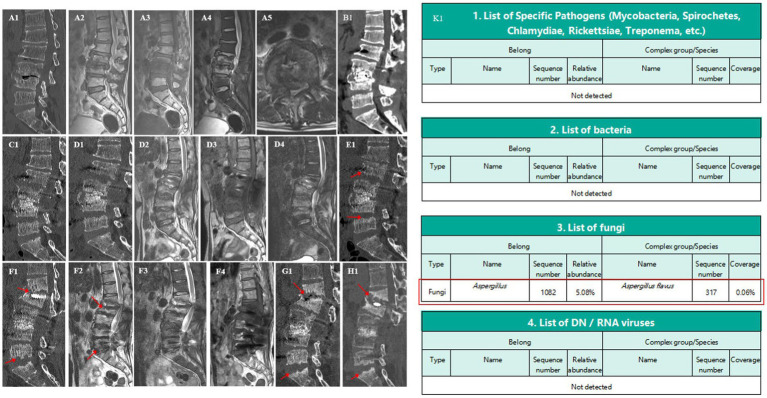
Spine CT/MRI and metagenomic sequencing. A1: moth-eaten changes at L3–L4, affecting discs and endplates. A2–A5: severe spinal canal occupation and potential spondylitis at L3–L4. B1,C1: progressive erosion in L4, L5, L2, and S1 endplates. D1–D4: relieved spinal canal occupation at L3–L4. E1: slow erosion and changes at the inferior endplate of L1. F1,G1: collapse of L1 anterior column and spinal canal mild stenosis. H1: stability and controlled inflammation. K1: Detection of *A. flavus*.

The patient was initially treated empirically with Cefuroxime sodium (1,500 mg every 8 h), alongside a plan for long-term glycemic control. Despite these interventions, his inflammatory markers continued to rise (ESR: 115 mm/H, CRP: 64.15 mg/L). CT imaging showed moth-eaten changes in the L3–L4 vertebral regions, affecting the intervertebral disc and vertebral body endplate (Figure 1A1). MRI investigations suggested severe spinal canal occupation at the L3–L4 segment and potential spondylitis (Figure 1A2–A5), and a biopsy revealed proliferative and degenerative changes without infectious agents. Considering infection, severe spinal canal occupation, moth-eaten changes in L3–L4 vertebral bodies, and possible late spinal instability, surgical intervention was pursued. This included posterior resection of the L3–L4 disc, removal of lesion tissue, laminectomy, spinal canal expansion, decompression, autologous bone grafting, and fixation with rods and screws extending from L2 to L5, accompanied by irrigation and drainage. Postoperative pathology confirmed chronic inflammation but no infectious agents. Linezolid (200 mg every 12 h) was added to the treatment regimen. Two weeks post-surgery, CT showed erosion at the L4 and L5 endplates (Figure 1B1). The patient was discharged on 8 April with reduced inflammation markers (ESR: 62 mm/H, CRP: 26.71 mg/L), improved lower back pain and lower limb symptoms, and good glycemic control. Five weeks post-surgery, a CT scan revealed further damage to the L4 and L5 endplates; meanwhile, similar changes in the L2 and S1 endplates were also observed (Figure 1C1).

Two months post-surgery (May 17, 2022), the patient returned with worsened lower back pain, right inner thigh pain, and high inflammation markers (ESR: 76 mm/H, CRP: 40.41 mg/L). In this admission, initial treatment with vancomycin and linezolid was ineffective, leading to a switch to piperacillin-tazobactam combined with linezolid; however, inflammation markers remained high. Further MRI and CT examinations showed a significant improvement in spinal canal occupation at the L3–L4 segment (Figure 1D1–D4). However, the intervertebral disc signals between L2–L3, L4–L5, and L5–S1 were abnormal, indicating erosion. The previously eroded endplates of L2, L3, L4, and L5 were worsening. A biopsy and metagenomic next-generation sequencing of the L2–L3 lesion yielded negative results. Treatment was adjusted to include vancomycin and meropenem, yet symptoms persisted. A further emission CT scan showed that L3–L4 metabolism was the most active. Lesion removal, biopsy, irrigation, and drainage were performed under percutaneous endoscopy. Subsequently, metagenomic next-generation sequencing finally identified *A. flavus*, with therapy switched to voriconazole (200 mg every 12 h) (Figure 1K1). This targeted anti-infective therapy led to gradual improvements of pain, with ESR and CRP levels decreasing to 51 mm/H and 32.95 mg/L, respectively. A lumbar spine CT scan showed slowed progression in the erosion of the most vertebral endplates after 3 months of internal fixation; however, moth-eaten changes began to appear on the inferior endplate of L1 (Figure 1E1). The patient was discharged on 29 July with significant relief of back pain and right inner thigh pain; glycemic control was also good and treatment was changed to oral voriconazole (200 mg every 12 h). Follow-up revealed an ESR of 19 mm/H and a CRP level of 2.80 mg/L. Follow-up visits showed stable imaging and inflammation levels after 6 and 9 months of internal fixation.

After 11 months of internal fixation, the patient was returned to the outpatient department due to back pain caused by carrying objects. MRI and CT scans indicated mild spinal canal stenosis at the L1–L2 level, with mild collapse of the L1 anterior column (Figure 1F1–F4). The L1–L2 segment showed obvious kyphosis, the Cobb angle had increased, and inflammatory markers had risen (ESR: 38 mm/H, CRP: 24.43 mg/L). After 14 months of internal fixation, lumbar spine CT showed a slow and limited progression of the vertebral endplate erosion from the inferior endplate of L2 to the superior endplate of S1, without further spread to the thoracic spine. However, the anterior column of the L1 vertebral body showed obvious collapse, kyphosis, and spinal instability (Figure 1G1). The medication for the patient was adjusted to terbinafine hydrochloride (250 mg every 24 h), taken orally by himself. After 18 months of internal fixation (August 26, 2023), the patient showed basic symptom relief, effective inflammation control (ESR: 12 mm/H, CRP: 7.40 mg/L), good fusion of the L3–L4 bone graft area, and significant glycemic management (Figure 1H1); however, the collapse of the L1 anterior column was still continuing. At the latest follow-up on November 28, 2023, the ESR was 14 mm/H and the CRP was 8.15 mg/L.

## Literature review and discussion

*A. flavus*, an atypical pathogen in spinal infections, shows a significant propensity for recurrent infections to infect immunocompromised individuals, particularly those with diabetes mellitus and post-transplant patients. Our review (1994–2023) of typical *A. flavus* spinal infection cases, as detailed in Table 1, reveals a higher incidence of this disease in individuals with weakened immune systems, including post-transplant patients. The comprehensive review by Gabrielli et al. (17) offers an insightful perspective on osteomyelitis caused by *Aspergillus* species. It highlights the prevalence of *A. flavus* infections in specific regions. Their study showed the relative commonness of *Aspergillus* osteomyelitis in some areas and offered a richer understanding of these infections in the broader epidemiological context. However, its evolving epidemiology calls for more vigilance and understanding in spinal infections. Fungal spinal infections often affect intervertebral discs and endplates, likely due to their limited blood supply and avascular nature, which hinder immune cell access and increase vulnerability to infection (18). The reliance of intervertebral discs on nutrient diffusion from vertebral bodies may also impede their ability to combat infections (19). This case, though exhibiting characteristics similar to those documented in the existing literature, revealed a noteworthy specificity. The fungal infection was confined exclusively to the lumbar segment. It primarily affected the intervertebral disc and endplate, and, significantly, did not extend to the thoracic spine. This unreported phenomenon is likely attributed to variations in blood supply and anatomy. The lumbar region’s richer blood supply may facilitate pathogen colonization and growth. Additionally, the lumbar spine, enduring more mechanical stress and offering greater mobility than the thoracic spine, may be more prone to injury, potentially heightening infection risk. Another specificity of this case is the persistent collapse of the anterior column of L1, which may have been caused by a combination of factors, including trauma, osteoporosis, vertebral body erosion, lumbar spine physiology, and the absence of internal fixation. However, considering the increased risk of infection in the remaining spinal segments with another surgery, the absence of lower limb neurological symptoms, and our expectation of spontaneous fusion at the L1–L2 segment to stability, we decided against surgical intervention but continued brace protection. Therefore, the evolving nature of fungal spinal infections, especially those caused by *A. flavus*, presents significant diagnostic challenges that require close attention.

**Table 1:** Main literature reports on *Aspergillus flavus* spinal infection in recent years.

Cases	Diagnosis	Treatment	Outcome	Year	References
9	*Aspergillus* spondylodiscitis	Drugs	Cured	1994	(8)
1	*Aspergillus* spondylodiscitis	Drugs+surgery	Cured	2000	(9)
1	*Aspergillus flavus* epidural abscess and osteomyelitis	Drugs+surgery	Dead	2003	(10)
1	Aspergillus flavus spondylodiscitis	Drugs+surgery	Dead	2008	(11)
2	*Aspergillus* spondylodiscitis	Drugs+surgery	Cured	2010	(12)
1	*Aspergillus* osteomyelitis	Drugs	Cured	2011	(13)
1	*Aspergillus flavus* spondylodiscitis with epidural abscess	Drugs	Cured	2012	(14)
1	*Aspergillus* spondylodiscitis	Drugs+surgery	Cured	2013	(15)
1	Aspergillus flavus spondylodiscitis	Drugs	Cured	2023	(16)

Fungal spinal infections often result from hematogenous dissemination, lung lesions, direct inoculation, or surgical procedures (2, 5). However, the lack of specific signs in the patient’s initial lung CT scan and clinical presentation, along with the impact of antibiotics and imaging analysis, led us to rule out lung lesions and postoperative fungal infection, despite some inconsistencies in prior reports (20, 21). Considering the patient’s history of diabetes, COVID-19, acupuncture treatment, and the working environment, these elements arise as potential factors of infection. Diabetes mellitus not only increases susceptibility to rare infections but may also complicate the clinical trajectory of such infections (22). The link between COVID-19 and subsequent *Aspergillus* infection, potentially through post-viral immunosuppression, remains under-explored (23). Moreover, the risk of *Aspergillus* infection stemming from acupuncture and the specific working environment, potentially causing direct spinal invasion, warrants consideration. Although there is a high level of suspicion regarding these routes of infection, early diagnosis of fungal spinal infections remains challenging. *Aspergillus* spondylitis also lacks specific clinical features. According to the literature reviews published by Dai et al. (24) and Gamaletsou et al. (2), *Aspergillus fumigatus* appears to be the most common *Aspergillus* species responsible for spinal fungal infections, followed by *A. flavus*. Both *Aspergillus fumigatus* and *A. flavus* spinal infections commonly present with lower back pain, which may or may not be accompanied by fever. Additional symptoms can include spinal nerve compression and even paraplegia. These infections are more prevalent in men and often involve the lumbar spine, followed by the thoracic and cervical spine. On imaging, there are no relevant reports on the differences in spinal infections caused by the two *Aspergillus* species; the main diagnostic results still come from histopathological examination. It seems that the differences in spinal infections caused by *Aspergillus* species are not specifically mentioned or valued. *Aspergillus* can exhibit slow growth in culture, occasionally yielding false-negative results despite infection (25). However, the advantages of metagenomics next-generation sequencing (mNGS) include high throughput, high sensitivity, and efficiency, allowing for rapid and precise analysis of extensive genomic data (26). It is applicable to various sample types, providing broad bioinformatics insights, suitable for diversity studies and functional analysis (27). Additionally, mNGS can identify novel or rare microbes and is used in disease diagnosis and research. mNGS has been used for the diagnosis of various infectious diseases, including endocarditis, pneumonia, febrile neutropenia, osteoarticular infections, and infections in returning travelers (28). This novel tool is highly effective for identifying microbial DNA and RNA in blood and other clinical specimens, offering an unbiased approach to pathogen identification. Our case study illustrates the effectiveness of metagenomics NGS in overcoming these obstacles and accurately detecting such elusive pathogens in spinal diseases. While histopathological examination is more conclusive, its efficacy hinges on the quality and representativeness of the biopsy sample. Consequently, diagnosing *Aspergillus* spinal infection is challenging due to ambiguous transmission routes, nonspecific symptoms, and limitations of imaging and laboratory tests, further complicated by difficulties in obtaining and analyzing biopsy samples.

Voriconazole is the primary treatment for *A. flavus* spinal infections; however, the literature shows variable outcomes, necessitating tailored therapeutic strategies that account for individual patient factors, especially in diabetics (24). Our comparative examination of previous reported cases underscores the substantial variability in both clinical presentations and responses to treatment. In our case, alongside surgical debridement and continued antifungal therapy with voriconazole, glycemic control is another crucial therapeutic factor. Moreover, it was found that voriconazole’s efficacy against *A. flavus* depended on drug exposure and the isolate’s minimum inhibitory concentration (MIC), with lower exposures required for strains with higher MICs, highlighting the need for precise dosing and drug selection in clinical practice (29). The challenges in diagnosing *A. flavus* were compounded by the patient’s diabetic status and the pathogen’s subtle presentation, underscoring findings from the literature that highlight the importance of a comprehensive, multifaceted diagnostic approach. Terbinafine hydrochloride, primarily used for skin fungal infections, effectively controlled clinical symptoms and inflammation indicators in our case after the patient self-switched to this medication. Given its initial effectiveness, we speculate whether it could be used for fungal spinal infections, similar to Voriconazole, although more clinical evidence is needed. This case aligns with reported trends and adds to the growing body of evidence on the complexity of managing such infections, especially in diabetic patients.

In summary, these strategies are crucial for effectively navigating the unique challenges posed by *A. flavus* and similar pathogens, thereby ensuring optimal patient outcomes in complex clinical scenarios. The specific nature of *A. flavus* infections, coupled with their often-elusive presentation, mandates a multifaceted diagnostic approach. Advanced diagnostic techniques such as metagenomic next-generation sequencing have shown promise in identifying these pathogens with greater precision and speed, thereby facilitating earlier intervention and improved management outcomes. The varied clinical presentations and treatment responses observed in *A. flavus* spinal infections underscore the importance of a patient-centric approach that takes into account the patient’s overall health, existing comorbidities, and the specific characteristics of the infection. To effectively manage *A. flavus* and similar pathogens, we suggest that once inflammatory changes in the intervertebral disc are detected, it is recommended to conduct comprehensive imaging examinations (such as enhanced MRI) and laboratory tests (such as blood cultures and antibody and antigen testing). Lesion biopsy and, if necessary, open surgical bone biopsy (including bacterial culture, fungal culture, and metagenomic next-generation sequencing, repeatedly) should also be performed. For cases of vertebral instability accompanied by neurological dysfunction and kyphotic deformity, or those without neurological dysfunction but with anticipated vertebral collapse leading to kyphotic deformity, it is recommended to undertake early decompression, bone grafting, fusion, and internal fixation, combined with antifungal medication (continued for 6 weeks to 3 months). Additionally, a comprehensive management plan that addresses both the infection and underlying conditions, such as diabetes, is crucial.

## Conclusion

*A. flavus* spinal infection is a rare, often low diagnostic yield, recurrent opportunistic infection, predominantly occurring in individuals with compromised immune systems. It necessitates early recognition and individualized treatment approaches due to its unique characteristics and challenges.

## Data availability statement

The original contributions presented in the study are included in the article/supplementary material, further inquiries can be directed to the corresponding author.

## Ethics statement

Written informed consent was obtained from the individual(s) for the publication of any potentially identifiable images or data included in this article.

## Author contributions

HL: Writing – original draft, Writing – review & editing. HP: Data curation, Writing – original draft. YL: Data curation, Writing – original draft. HW: Data curation, Resources, Visualization, Writing – review & editing. SL: Investigation, Project administration, Supervision, Writing – review & editing. CX: Formal analysis, Funding acquisition, Project administration, Resources, Writing – original draft.
